# A network analysis of depressive and internet addiction symptoms among bullying-victimized adolescents: a comparison between left-behind and non-left-behind children

**DOI:** 10.3389/fpsyt.2026.1775940

**Published:** 2026-04-28

**Authors:** Qi Li, Jiannan Zheng, Zhijie Yan, Ruifeng Liu

**Affiliations:** 1 School of Physical Education, Central China Normal University, Wuhan, China; 2 School of Physical Education, Hubei University, Wuhan, China; 3 Sports Coaching College, Beijing Sport University, Beijing, China

**Keywords:** depressive symptoms, internet addiction, left-behind children, network analysis, suffering from school bullying

## Abstract

**Objective:**

School bullying is an important risk factor for mental health problems in adolescents. Depressive symptoms and internet addiction often co-occur among adolescents who have experienced bullying; however, the associations among specific symptoms remain unclear. This study aimed to investigate the comorbidity network characteristics of depressive symptoms and internet addiction among bullying-victimized adolescents, and to compare differences in network characteristics between left-behind and non-left-behind adolescents.

**Methods:**

This study employed a cross-sectional design and included 15,984 bullying-victimized adolescents selected through stratified random sampling. Depressive symptoms were assessed using the Center for Epidemiologic Studies Depression Scale (CES-D), and internet addiction was measured using the 9-item Internet Addiction Scale. A mixed graphical model (MGM) was used to construct the symptom network of depressive symptoms and internet addiction. Node centrality and bridge centrality indices were calculated, and network stability was assessed using bootstrap methods. The Network Comparison Test was conducted to compare differences in network structure and global strength between left-behind and non-left-behind adolescents.

**Results:**

Among bullying-victimized adolescents, the overall predictability of network nodes was 0.27. Risky persistence and Tolerance showed the highest strength centrality in the symptom network. The principal bridge symptoms were Risky persistence, Escape coping, and Depressive affect. The network model demonstrated good stability. No significant differences were observed between left-behind and non-left-behind adolescents in terms of either the overall network structure or global strength (all P > 0.05).

**Conclusion:**

Compared with interventions targeting only inter-symptom relationships, exposure to school bullying itself may be a more critical and primary intervention target. Left-behind and non-left-behind children exhibited highly similar symptom network patterns; therefore, intervention strategies should not rely solely on identity-based distinctions, but rather focus on bullying exposure and symptom severity to implement more precise interventions. These findings provide symptom-level empirical evidence for developing integrated intervention strategies for adolescents exposed to school bullying.

## 1 Introduction

School bullying is a serious public health issue that is prevalent among adolescents worldwide, with approximately one-third of adolescents globally having been exposed to bullying situations in school settings (1, 2). School bullying refers to intentional and repeated behaviors in which an individual or group inflicts harm on another individual who is in a disadvantaged position in terms of physical or psychological power (3). It typically involves two aspects: victimization and perpetration. Existing research indicates that perpetrators, victims, and those who simultaneously occupy both roles may all experience adverse mental health outcomes (4). Compared with the other two roles, bullying-victimized adolescents may face more pronounced negative consequences, including elevated depressive symptoms, internet addiction, and functional impairment (5).

Depressive symptoms and internet addiction represent two important dimensions of adolescents’ emotional distress and behavioral maladjustment. Previous research has shown that exposure to school bullying not only significantly increases the risk of depressive symptoms among adolescents but is also closely associated with elevated levels of internet addiction (6).According to the multi-circuit neuronal hyperexcitability (MCNH) hypothesis of mental disorders (7), internet addiction disorder and various psychiatric conditions may share dysregulation across multiple key neural circuits. This theoretical perspective suggests that, among bullying-victimized adolescents, internet addiction and depressive symptoms may not exist in isolation but may co-occur or exhibit closely interconnected symptom patterns. Therefore, it is necessary to further examine, at the symptom level, the associations between depressive symptoms and internet addiction among bullying-victimized adolescents, in order to better understand their patterns of co-occurrence and to inform targeted interventions.

From the perspective of traditional psychopathology, adolescents’ depressive symptoms and internet addiction are typically regarded as being driven by an underlying latent disorder or shared etiological factors (8, 9). Consequently, many studies have relied on the total scores of standardized scales to represent the severity of these conditions. However, such approaches may obscure the heterogeneity among individual symptoms and the genuine interactions that exist between them (10). Network analysis offers a promising alternative for understanding the complex and dynamic relationships among psychological symptoms (9, 10). This approach conceptualizes mental disorders as systems composed of interacting symptoms, in which different symptoms form complex network structures through direct associations (11). Within this framework, the emergence and persistence of symptoms are not solely driven by a common underlying cause but are maintained through a series of mutual interactions among symptoms (12). Under the framework of network psychopathology, specific mental disorders are modeled as networks consisting of nodes and edges, where nodes represent individual symptoms and edges reflect conditional dependency relationships between symptoms (12, 13). Some symptoms may function as bridge symptoms, linking the symptom networks of different mental disorders (14, 15). Therefore, this approach not only helps reveal patterns of interaction at the symptom level but also provides important methodological support for understanding the mechanisms underlying comorbidity between different mental disorders. Several studies have applied the network analytic framework to investigate the comorbidity between depressive symptoms and internet addiction among adolescents. For example, a survey conducted among Chinese adolescents found that “Could not get things going,” “Felt sadness,” and “Reluctant to be offline” were central symptoms within the comorbidity network of depressive symptoms and internet addiction (16). Another cross-sectional study conducted in Macao, China during the COVID-19 pandemic, using the self-report Internet Addiction Test (IAT) and the 9-item Patient Health Questionnaire (PHQ-9), reported that “Preoccupation with the Internet,” “Neglect chores to spend more time online,” “Guilty,” and “Request an extension for longer time spent online” were the most central symptom nodes in the comorbidity network of adolescents’ depressive symptoms and internet addiction (17).

Although bullying-victimized adolescents often experience more pronounced negative outcomes in terms of depressive symptoms and internet addiction, previous studies have shown that daily supervision, emotional support, and value guidance provided by families remain important protective factors for promoting psychological adjustment in this population (18–20). Influenced by the uneven economic development between urban and rural areas and across regions in China, many parents migrate to economically developed areas for employment in pursuit of higher income. Their children are typically cared for by one parent or other relatives and are commonly referred to as left-behind children (21). Compared with non-left-behind children, left-behind children often face prolonged parental absence, limited parent–child communication, and changes in daily caregiving structures. Consequently, their sources of emotional support and strategies for coping with stress may exhibit distinct characteristics (22–24). For bullying-victimized adolescents, such developmental contextual differences may not only influence the manifestation of psychological problems but may also alter the connections between depressive symptoms and internet addiction symptoms, thereby leading to differences in the centrality and bridging roles of specific symptoms within the comorbidity network.

Although this analytical strategy has been applied in general adolescent populations and other clinical groups (25–27), bullying-victimized adolescents face unique mental health challenges, and the clinical characteristics of their depressive symptoms and internet addiction may differ substantially (28, 29). Therefore, it is of great significance to delineate the comorbidity structure of depressive symptoms and internet addiction symptoms among adolescents who have experienced school bullying using network analysis. Based on the above, the present study adopted a cross-sectional design and employed network analysis to examine the comorbidity structure of depressive symptoms and internet addiction symptoms among bullying-victimized adolescents. Specifically, the aims of this study were to: (1) describe the network structure of depressive symptoms and internet addiction symptoms among bullying-victimized adolescents; (2) identify the core symptoms in the network as well as the bridge symptoms linking the two symptom communities; and (3) compare differences in network structure and global connectivity between left-behind and non-left-behind bullying-victimized adolescents. This was an exploratory study that aimed to reveal the patterns of association between different psychological problems at the symptom level and to provide a reference for subsequent mechanistic research.

## 2 Methods

### 2.1 Study design and participants

This study employed a cross-sectional design. All data were collected between September and November 2024 from 17 prefecture-level cities in Hubei Province using a stratified sampling method. In each prefecture-level city (or autonomous prefecture), seven schools were selected, including four urban schools (two junior high schools and two senior high schools) and three county-level schools (two junior high schools and one senior high school). Within each school, grades were used as strata and classes as the sampling units. Intact classes were randomly selected from each grade, with at least approximately three classes (about 240 students) sampled from each school. If the number of students in a school was insufficient, additional samples were recruited from schools of the same type within the same area to ensure adequate sample sizes across all strata.

Bullying victimization was assessed using the revised Olweus Bully/Victim Questionnaire (30). A total of 20,012 participants were initially included. We excluded individuals who reported never having used the Internet (n = 3,986) and those with incomplete information on depressive symptoms or internet addiction (n = 94). Ultimately, 15,984 participants were included in the present analysis. **Inclusion criteria:1.**Students who participated in the Hubei Provincial Student Common Diseases and Health Influencing Factors Surveillance Program.2.Students who completed the revised Olweus Bully/Victim Questionnaire. **Exclusion criteria:**1.Individuals lacking information on bullying victimization or with clearly incomplete questionnaire responses.2.Students who reported never having used the Internet, as internet addiction symptoms could not be assessed.3.Participants with missing or incomplete information on depressive symptoms or internet addiction.

### 2.2 Ethical considerations

This study was part of the Hubei Provincial Student Common Diseases and Health Influencing Factors Surveillance Program. The study protocol was approved by the Ethics Committee of the Hubei Provincial Center for Disease Control and Prevention (Approval No. 2024-016-01) and was conducted in accordance with the ethical principles of the Declaration of Helsinki. Data were collected by professionally trained staff. After data collection, designated personnel from the county-, municipal-, and provincial-level Centers for Disease Control and Prevention were responsible for data entry, storage, and stepwise reporting. To ensure data quality, approximately 5% of students at each survey site were randomly selected for on-site verification. All data underwent multilevel review, validation, and reporting through the surveillance system. Before being provided to the research team, all datasets were thoroughly anonymized by the surveillance management department and contained no personally identifiable information. Therefore, the ethics committee waived the requirement for written informed consent.

### 2.2 Measurement tools

Depressive symptoms were assessed using the Center for Epidemiologic Studies Depression Scale (CES-D) (31), which consists of 20 items, such as “I was bothered by things that usually do not bother me” and “I did not feel like eating; my appetite was poor.” All items were rated on a 4-point scale (0 = less than 1 day, 1 = 1–2 days, 2 = 3–4 days, 3 = 5–7 days). The scale has been widely validated in Chinese samples and has demonstrated good structural stability and psychometric properties (32, 33).Internet addiction (IA) was measured based on the relevant criteria of the Diagnostic and Statistical Manual of Mental Disorders, Fifth Edition (DSM-5) (34), with revisions made with reference to the Internet Addiction Scale developed by Tao et al. (35). This scale includes 9 items with dichotomous responses (1 = yes, 2 = no). A sample item is: “I have repeatedly tried to stop going online, but I am never able to control myself.” This scale is the designated instrument for the National Student Common Diseases and Health Influencing Factors Surveillance Program in China. It has been widely used in surveillance surveys across multiple provinces and has demonstrated good reliability and validity (36–39). In the present sample, the internal consistency of the scale was acceptable, with a Kuder–Richardson coefficient (KR-20) of 0.78.School bullying was assessed using six items from the revised Olweus Bully/Victim Questionnaire (30). The questionnaire covers verbal bullying, relational bullying, and physical bullying occurring in the past 30 days. Items were rated on a 3-point scale (0 = never, 1 = sometimes, 2 = often). Participants who selected “sometimes” or “often” on any item were classified as victims of bullying. This instrument has been validated and widely used in research on Chinese adolescents, with good reliability and validity (40–42). Left-behind status was determined based on participants’ self-reported questionnaire responses. A sample question was: “Who have been living with you during the past six months?” Based on definitions commonly used in previous studies (27), adolescents who had been cared for by a single parent or other relatives for more than six consecutive months due to one or both parents migrating for work were defined as left-behind children.

### 2.3 Statistical analysis

Before conducting the network analysis, it was necessary to address potential redundancy among scale items. Previous research has indicated that substantial content overlap between items may lead to redundancy and local dependence, which can artificially inflate connections among depressive symptoms and affect the interpretation of the network structure (43). The CES-D scale contains 20 items that reflect several core dimensions of depression, resulting in notable content overlap and structural clustering. Prior studies have widely supported the four-factor structure of the CES-D across different populations. However, studies involving Chinese and other Asian adolescent samples have found that a three-factor structure may demonstrate superior or comparable model fit, suggesting that the factor structure of the CES-D may vary across cultural contexts (44, 45). In light of this evidence, and to balance measurement structure with the characteristics of the present sample, the current study adopted a three-factor structure to aggregate CES-D items.

#### 2.3.1 Network analysis model

The network analytic model was primarily constructed using the bootnet package (version 1.6) and the qgraph package (version 1.9.8) in R (version 4.5) (14). In the network, symptoms assessed by the scales were defined as nodes, and the associations between these symptoms were defined as edges (46). Based on previous theoretical and empirical work, the present study included 12 symptom nodes:(1) The 20 items of the Center for Epidemiologic Studies Depression Scale (CES-D) were grouped into three dimensions: Depressive affect (items 3, 6, 9, 10, 14, 15, 17, 18, and 19), Positive affect (items 4, 8, 12, and 16), and Somatic symptoms (items 1, 2, 5, 7, 11, 13, and 20), with the four items of Positive affect reverse coded (14).(2) The nine items assessing internet addiction (IA) were: Preoccupation, Withdrawal, Tolerance, Loss of interest, Loss of control, Role impairment, Concealment, Risky persistence, and Escape coping. Because the scale is typically conceptualized as having a single-factor structure, each item represents a distinct addiction symptom. Within the network analysis framework, symptoms are treated as network nodes, and retaining the original items helps reveal the interrelationships among individual symptoms.

A Mixed Graphical Model (MGM) algorithm was used to construct the network. This algorithm was implemented using the mgm package (version 1.2.15) in R (47), which is suitable for estimating networks with mixed data types (48). Edge sparsity was controlled using Least Absolute Shrinkage and Selection Operator (LASSO) regularization, and the Extended Bayesian Information Criterion (EBIC) was used to optimize the balance between model fit and model complexity. The EBIC hyperparameter γ was set to 0.5 to balance model fit and network sparsity, thereby selecting the optimal connectivity structure for the entire network (49, 50). The resulting pairwise weighted adjacency matrix was visualized using the Fruchterman–Reingold force-directed algorithm (49). In the network graph, the thickness of edges represents the strength of the associations between nodes; green solid edges indicate positive associations, whereas red dashed edges indicate negative associations. Node centrality indices reflecting the relative importance of nodes within the overall network structure—Strength, Closeness, Betweenness, and Expected Influence—were calculated and visualized using the centrality Plot function (14). In addition, to explore cross-community transmission mechanisms between internet addiction and depressive symptoms (11), the networktools package (version 1.6.0) was used to estimate bridge centrality metrics (e.g., bridge strength, bridge closeness, and bridge betweenness), which identify bridge symptoms that play key roles in linking the two symptom communities (25, 26).

#### 2.3.2 Evaluate the stability of the network model

To evaluate the accuracy and stability of the network structure, the bootnet package was used to perform nonparametric bootstrap and case-dropping bootstrap procedures. The case-dropping bootstrap (1,000 iterations) was conducted by progressively removing portions of the sample and re-estimating the network to calculate the Correlation Stability Coefficient (CS-coefficient). The CS-coefficient reflects the correlation between the centrality indices obtained from the reduced samples and those from the original sample. According to recommended criteria, CS ≥ 0.25 indicates the minimum acceptable level of stability, whereas CS ≥ 0.50 indicates good stability and supports reliable interpretation of the network results (15).

#### 2.3.3 Heterogeneity of symptom networks between left-behind and non-left-behind groups

The NetworkComparisonTest package (version 2.2.2) in R was used to assess differences in network characteristics between left-behind and non-left-behind groups (14). First, the overall invariance of network structure and strength was examined by comparing the distribution of edge weights across the two networks and the average difference in global strength. Second, edge-level comparisons were conducted to test differences in individual edge weights between the two groups, with the significance level set at P < 0.05 (two-tailed). The Holm–Bonferroni method was applied to adjust p-values in order to control the Type I error rate and reduce the risk of false-positive findings.

## 3 Results

### 3.1 Description of the participating samples

A total of 15,984 participants were included in the present study (mean age = 14.33 ± 1.68 years), including 7,141 boys (44.7%) and 8,843 girls (55.3%). **Table 1** presents the positive response rates for each internet addiction item and the mean scores (± standard deviations) for each dimension of depressive symptoms among the participants.

**Table 1:** Participant baseline characteristics.

Variable	Total (N = 15984)	Left-behind children	
		Yes	No
Age	14.33 ± 1.68	14.21 ± 1.66	14.39 ± 1.68
Gender			
Female	8843(55.3)	3006(55.4)	5837(55.3)
Male	7141(44.7)	2418(44.6)	4723(44.7)
Grade			
Junior High School	9177(57.4)	3331(61.4)	5846(55.3)
Senior High School	5762(36)	1833(33.8)	3929(37.2)
Vocational High School	1045(6.6)	260(4.8)	785(7.4)
Internet addiction			
IA1	5770(36.1)	2035(37.5)	3735(35.4)
IA2	2715(17.0)	971(17.9)	1744(16.5)
IA3	4138(25.9)	1485(27.4)	2653(25.1)
IA4	1693(10.6)	601(11.1)	1092(10.3)
IA5	3933(24.6)	1354(25.0)	2579(24.4)
IA6	763(4.8)	259(4.8)	504(4.8)
IA7	1101(6.9)	407(7.5)	694(6.6)
IA8	2910(18.2)	1047(19.3)	1863(17.6)
IA9	5353(33.5)	1945(35.9)	3408(32.3)
Depressive symptoms			
Depressive affect	15.86 ± 6.03	16.22 ± 6.16	15.67 ± 5.96
Positive affect	10.20 ± 3.32	10.49 ± 3.28	10.04 ± 3.33
Somatic symptoms	12.38 ± 4.25	12.59 ± 4.33	12.27 ± 4.20

Continuous variables are presented as mean ± standard deviation; categorical variables are expressed as number (percentage). For each item of Internet Addiction (IA), the response count and response rate are based on the “Yes” option. IA1 = Preoccupation; IA2 = Withdrawal; IA3 = Tolerance; IA4 = Loss of interest; IA5 = Loss of control; IA6 = Role impairment; IA7 = Concealment; IA8 = Risky persistence; IA9 = Escape coping.

### 3.2 Construction of a comorbidity network between depressive symptoms and internet addiction

**Figure 1:**
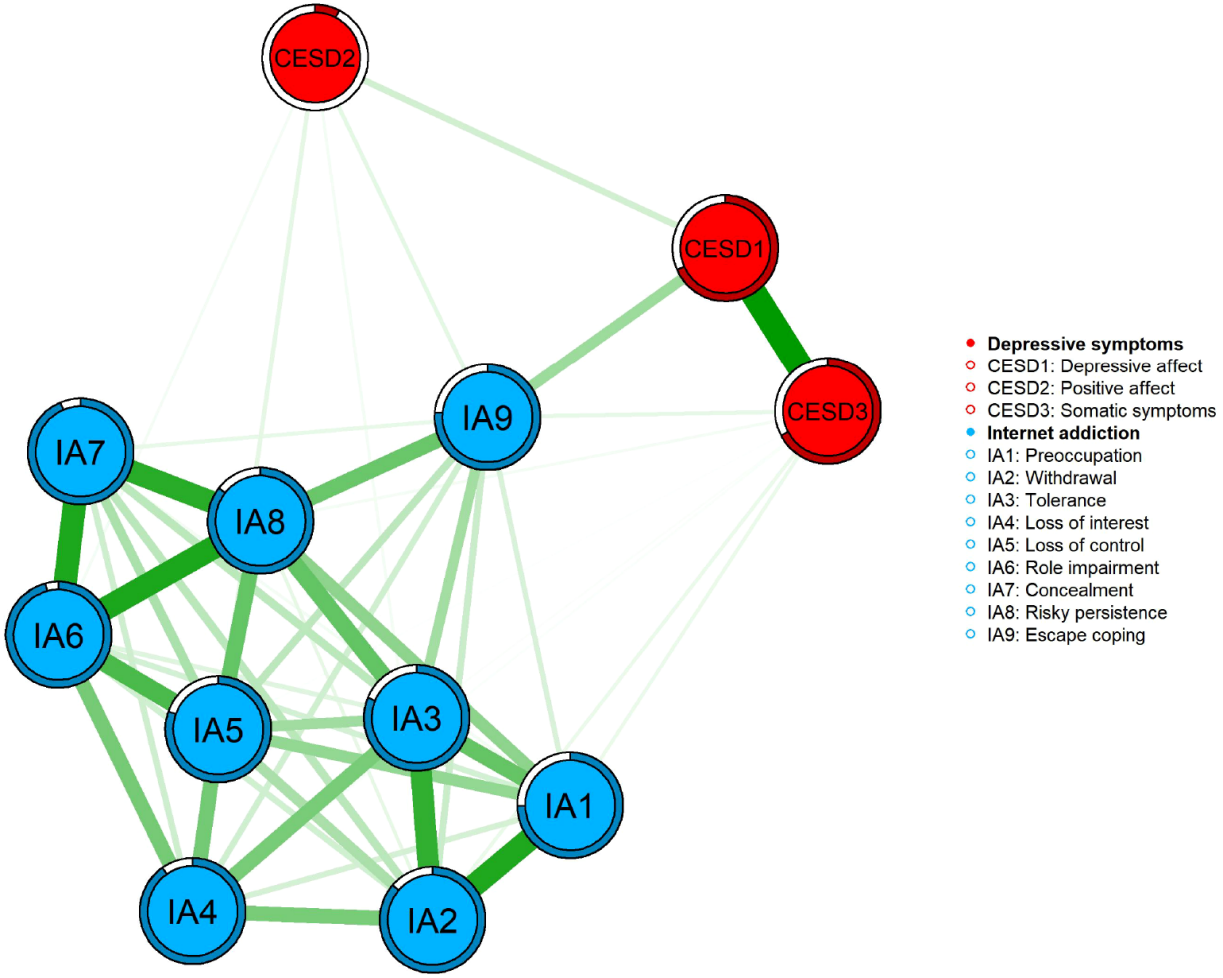
Estimated network model linking depressive symptoms and Internet addiction among adolescents(n = 15984).

**Figure 2:**
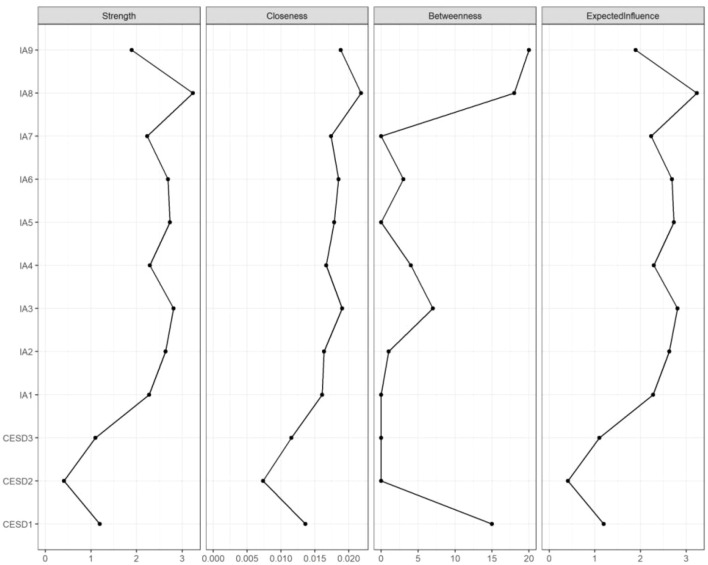
The centrality index of the network linking adolescent depressive symptoms and internet addiction. IA, internet addiction; CESD, depressive symptoms. IA1–IA9 denote Preoccupation, Withdrawal, Tolerance, Loss of interest, Loss of control, Role impairment, Concealment, Risky persistence, and Escape coping, respectively. CESD1–CESD3 denote Depressive affect, Positive affect, Somatic symptoms, respectively.

**Figure 1** presents the visualized comorbidity network of internet addiction and depressive symptoms among bullying-victimized adolescents, constructed using the MGM model. The predictability of each symptom node is illustrated by ring-shaped pie charts surrounding the nodes. The average predictability of symptom nodes in the sample was 0.27, indicating that, on average, 27% of the variance of each node could be explained by its neighboring nodes in the network. Among the depressive symptom nodes, CESD1 (Depressive affect) and CESD3 (Somatic symptoms) showed relatively high predictability, with values of 0.68 and 0.60, respectively, which were higher than those of other nodes in the model. Within the network, the strongest association was observed between CESD1 (Depressive affect) and CESD3 (Somatic symptoms) (r = 0.76). Strong associations were also identified between IA6 (Role impairment) and IA7 (Concealment), as well as between IA6 (Role impairment) and IA8 (Risky persistence) (r = 0.66). In addition, a relatively strong connection was observed between IA7 (Concealment) and IA8 (Risky persistence) (r = 0.63).The results of the network stability analysis are presented in **Supplementary Figures S1**, **S2**. The case-dropping bootstrap analysis indicated that strength centrality demonstrated good stability (CS-coefficient = 0.75). In addition, the 95% confidence intervals obtained from the nonparametric bootstrap procedure suggested that the edge-weight estimates were sufficiently accurate. **Figure 2** presents the centrality indices of the network. Descriptive results indicate that IA8 (Risky persistence) and IA3 (Tolerance) exhibited relatively high strength centrality within the network.

**Figure 3:**
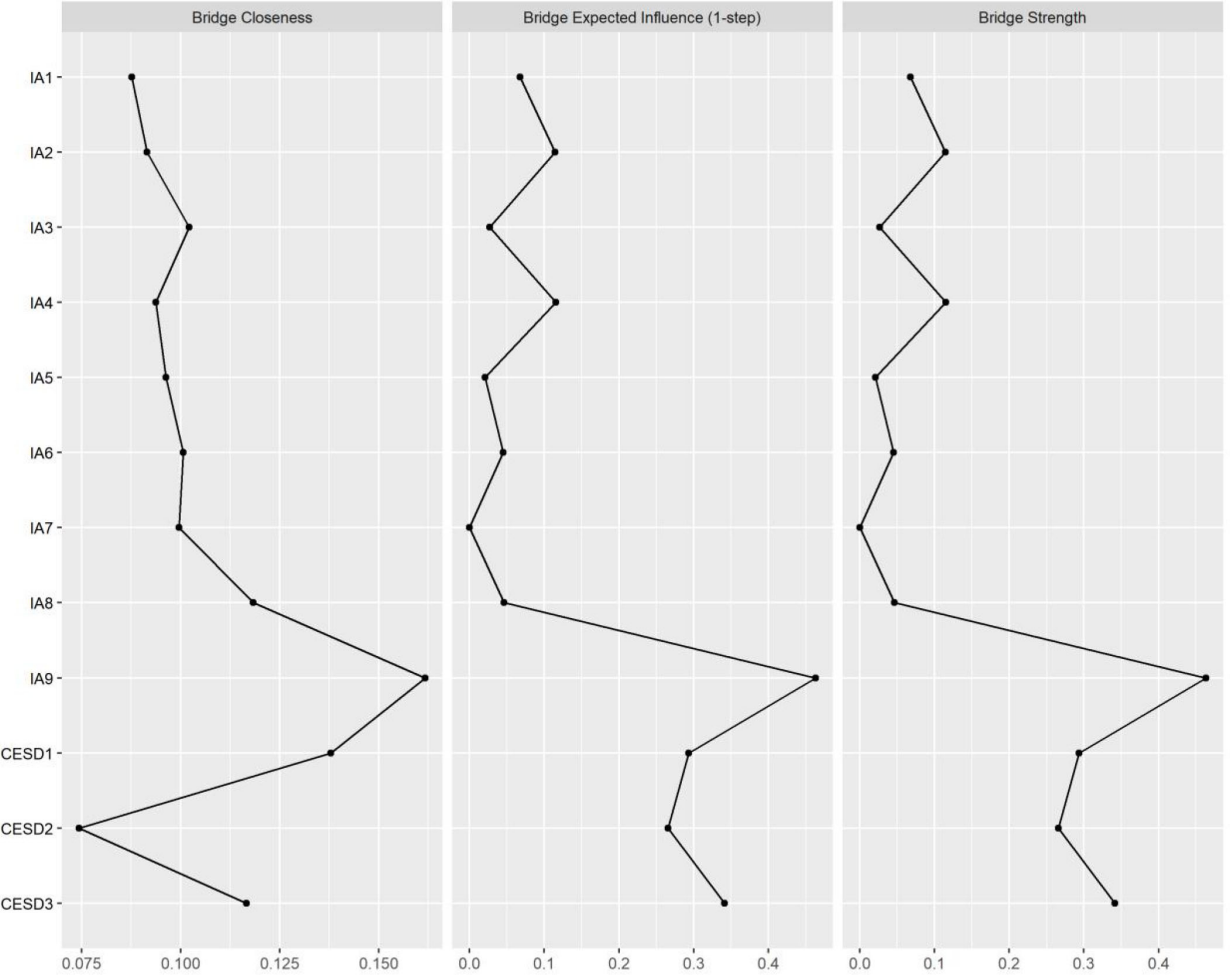
Bridging centrality indicators of adolescent depressive symptoms and internet addiction networks. IA, internet addiction; CESD, depressive symptoms. IA1–IA9 denote Preoccupation, Withdrawal, Tolerance, Loss of interest, Loss of control, Role impairment, Concealment, Risky persistence, and Escape coping, respectively. CESD1–CESD3 denote Depressive affect, Positive affect, Somatic symptoms, respectively.

**Figure 3** displays the bridge centrality results for the network of internet addiction and depressive symptoms. IA8 (Risky persistence), IA9 (Escape coping), and CESD1 (Depressive affect) showed relatively high bridge strength, suggesting that these nodes may play prominent connecting roles in linking the two symptom communities. The stability of the bridge nodes was also found to be satisfactory (see **Supplementary Figure 3**).

### 3.3 Network comparison between left-behind and non–left-behind children

After comparing the network characteristics between the left-behind and non-left-behind groups (see **Supplementary Figures S4**, **S5**), no significant differences were found in the overall network structure (M = 0.052, p = 0.383) or global strength (S = 0.032, p = 0.607).In the edge-level comparisons, a small number of edges reached statistical significance before multiple comparison correction; however, these associations were no longer significant after applying the Holm–Bonferroni correction. These findings suggest that there were no stable between-group differences in the network structure or overall connectivity between the two groups.

## 4 Discussion

This study is the first to construct a comorbidity network of depressive symptoms and internet addiction in a large cross-sectional sample of bullying-victimized adolescents, and to further compare differences in network characteristics between left-behind and non-left-behind groups. The main findings of the present study can be summarized as follows. First, among bullying-victimized adolescents, internet addiction and depressive symptoms exhibited a certain degree of co-occurrence. However, subsequent analyses indicated that the overall predictability of the symptom network was only 27%, suggesting relatively low interdependence among symptoms. Second, within the depressive symptom cluster, a strong association was observed between CESD1 (Depressive affect) and CESD3 (Somatic symptoms). Within the internet addiction symptom cluster, relatively strong connections were observed among IA6 (Role impairment), IA7 (Concealment), and IA8 (Risky persistence). These findings suggest that these symptoms remained strongly connected even after controlling for other symptoms in the network and may form tightly interconnected structures or local symptom clusters within their respective symptom communities. Third, IA8 (Risky persistence) and IA3 (Tolerance) showed relatively high strength centrality, whereas IA8 (Risky persistence), IA9 (Escape coping), and CESD1 (Depressive affect) exhibited relatively high bridge centrality. These results suggest that these symptoms may play important roles in maintaining the overall network and in linking the symptom systems of internet addiction and depression. Fourth, no significant differences were found between left-behind and non-left-behind groups in terms of overall network structure, global strength, or edge differences after correction, indicating that the overall symptom network patterns were largely similar between the two groups.

In the present sample of bullying-victimized adolescents, the overall node predictability of the depression–internet addiction comorbidity network was 27%, which is lower than the 46% reported in a study of general adolescents in Macao (17). Previous research has suggested that node predictability may vary substantially across different samples and even within the same network, and that clinical or high-risk populations often exhibit lower average predictability (51). Higher node predictability indicates stronger internal self-maintenance within the network, implying that interventions targeting key symptoms may be more effective in improving overall symptom functioning (52). In the present sample, the relatively low overall node predictability suggests that adolescents’ symptoms may be influenced to a greater extent by external factors outside the network, rather than being primarily maintained through interactions among symptoms within the network. Consequently, interventions focusing solely on internal network symptoms may have limited effectiveness. In contrast, directly addressing school bullying, the external risk factor underlying these difficulties, may represent a more critical and effective strategy. It should be noted that these findings are preliminary and exploratory, and further studies with larger samples and longitudinal designs are needed to validate these conclusions.

IA8 (Risky persistence) emerged as both a core symptom and a bridge symptom in the network model constructed in this study. This pattern may reflect the psychological vulnerability of bullying-victimized adolescents when confronted with stressful situations. On the one hand, individuals exposed to bullying victimization often experience persistent negative self-evaluations and emotional distress, which may increase their tendency to use the Internet as a means of avoidance or emotional regulation (53, 54). However, such compensatory Internet use is often associated with a higher risk of addiction, particularly when individuals already experience psychosocial difficulties prior to adopting this coping strategy. In such cases, the negative consequences of this coping pattern may be amplified, potentially forming a cycle of “negative emotions – excessive Internet use – worsening negative emotions” (55). On the other hand, this emotionally driven pattern of Internet use may weaken individuals’ ability to weigh long-term consequences, allowing risk-taking patterns of Internet use to occupy a more central position within the symptom network (56). IA3 (Tolerance), another core symptom identified in the network, together with IA8 (Risky persistence) may constitute a key dynamic mechanism within the internet addiction symptom system. Specifically, Tolerance reflects the increasing need for reinforcement, whereas Risky persistence reflects the behavioral tendency to continue Internet use despite negative consequences. The interaction between these two symptoms may facilitate the gradual shift of Internet use from an emotion regulation strategy to an uncontrolled behavioral pattern at the symptom level. Over time, this process may exacerbate individuals’ negative emotional experiences and physiological stress responses, thereby further reinforcing depressive symptoms.

Similar to IA8 (Risky persistence), IA9 (Escape coping) and CESD1 (Depressive affect) also showed relatively high bridge strength centrality. As a common consequence of school bullying (57), Depressive affect not only reflects individuals’ psychological distress but may also be closely related to the coping strategies they adopt. Escape coping, in turn, reflects a strategic tendency to alleviate negative experiences by avoiding real-life problems or turning to the online environment when facing stressful situations. In this process, individuals with elevated depressive affect may be more likely to adopt escape-oriented coping strategies, and this coping style may further increase their reliance on Internet use. As a result, these two symptoms may exhibit cross-community linkage at the symptom level, thereby contributing to the co-occurrence of depressive symptoms and internet addiction.

Based on prior research suggesting differences in family supervision, emotional support, and parent–child interaction, we originally expected that left-behind and non-left-behind bullying-victimized adolescents might differ in the linkage structure between depressive symptoms and internet addiction symptoms. However, the network comparison results showed that the two groups did not differ significantly in either overall network connectivity or the distribution of edge weights. This may suggest that, under the shared context of the strong stressor of school bullying, the interaction patterns within the symptom networks of the two subgroups are highly similar. In other words, the adverse developmental circumstances faced by left-behind children may not necessarily further alter the organizational structure of the symptom network itself.

More specifically, the vulnerability associated with left-behind status (58, 59) may not operate primarily by altering the patterns of connections among symptoms. Once symptoms emerge, left-behind adolescents may experience more rapid symptom deterioration and more severe functional impairment (60, 61). For example, prolonged parental absence, limited parent–child communication, and changes in caregiving arrangements may weaken adolescents’ emotional buffering capacity and stress-regulation resources after experiencing bullying (62, 63), making them more likely to enter a state of symptom activation even when exposed to a similar symptom network structure (64). In addition, the overall node predictability in the present sample was relatively low (r = 0.27), further suggesting that the onset and maintenance of symptoms may be driven more by factors external to the network. This finding contributes to a more precise understanding of the role of left-behind status in the psychological adjustment of bullying-victimized adolescents: its influence may primarily lie in shaping stress-coping resources, emotion regulation processes, and environmental buffering conditions, rather than directly altering the comorbidity structure between depressive symptoms and internet addiction symptoms (65). From an intervention perspective, for adolescents who have experienced school bullying, the focus should not be limited to symptom management alone. Greater attention should also be paid to the identification, prevention, and intervention of bullying experiences, as well as to strengthening family support, emotion regulation, and stress-coping resources. This may be especially important for left-behind children, whose real-life support systems are often more limited.

Although the present study provides useful symptom-level insights into the comorbidity of depression and internet addiction among bullying-victimized adolescents, several limitations should be acknowledged. First, this was a cross-sectional study, which precludes any inference of causal relationships among variables. Future research should adopt longitudinal designs to establish temporal sequences and clarify potential causal mechanisms. Second, all information in this study was obtained through self-report measures, and participants’ subjectivity may have introduced bias into the findings. Finally, although the network model demonstrated acceptable stability, its estimation remained constrained by the coverage of the measurement instruments and the insufficient control of potential confounding factors (e.g., family functioning and comorbid disorders). Future studies could further examine the robustness and external validity of the network structure by incorporating multi-informant data, more comprehensive measurement indicators, and relevant contextual variables.

## 5 Conclusion

Among bullying-victimized adolescents, compared with interventions that focus solely on the relationships among symptoms, external risk factors—particularly exposure to school bullying itself—may represent more critical and prioritized intervention targets. Second, several symptoms in the network, such as IA8 (Risky persistence) and IA3 (Tolerance), exhibited relatively high centrality, whereas IA8 (Risky persistence), IA9 (Escape coping), and CESD1 (Depressive affect) showed relatively high bridge centrality. These nodes may play important roles in maintaining the symptom network and linking different symptom communities. Therefore, intervention strategies should place particular emphasis on symptoms with high centrality and bridging functions, in order to improve intervention efficiency and disrupt potential pathways linking symptoms. Finally, because left-behind and non-left-behind adolescents showed similar symptom network patterns, interventions for left-behind children should not be differentially designed solely on the basis of their identity status. Instead, greater attention should be directed toward their exposure to bullying and the severity of specific symptoms, so that more targeted intervention measures can be implemented.

## 6 Strengths

This study has several notable strengths. To the best of our knowledge, it is the first study to construct a symptom-level comorbidity network of depressive symptoms and internet addiction in a large sample of bullying-victimized adolescents. Focusing on this high-risk population enhances the contextual ecological validity and practical relevance of the findings.

Second, this study employed a symptom network analytic approach to characterize the conditional associations and comorbidity structure between depressive symptoms and internet addiction symptoms at the symptom level, thereby providing useful evidence for identifying potential key symptoms and intervention targets.

Third, this study further compared differences in symptom network structure between left-behind and non-left-behind adolescents, thereby extending existing research on how psychological problems may manifest across different developmental contexts. Although no significant differences were identified, this finding provides empirical support for the possibility that symptom network structures may remain similar under a shared high-stress context, which has important implications for deepening our understanding of the mechanisms through which left-behind status may influence psychological adjustment.

## Data Availability

The raw data supporting the conclusions of this article will be made available by the authors, without undue reservation.
