# Supplementary material for: A network analysis of depressive and internet addiction symptoms among bullying-victimized adolescents: a comparison between left-behind and non-left-behind children
Source: Front Psychiatry. 2026 Apr 28;17:1775940. doi: 10.3389/fpsyt.2026.1775940 (PMC13161157; doi:10.3389/fpsyt.2026.1775940)
Supplement: Supplementary file 1 [file SupplementaryFile1.docx]

**Figure S1.**Stability test of network centrality index

**Figure S2.**Network edge weight stability test

**Figure S3.**Stability of network bridge strength

**Figure S4.**Estimated network model for the association between Internet addiction and depression in Left-Behind(N=5440) and Non–Left-Behind Children（N=10586）

**Figure S5.** Comparison of network centrality indices between Non–Left-Behind Children and Left-Behind Children.

**Note.** IA = internet addiction; CESD = depressive symptoms. IA1–IA9 denote Preoccupation, Withdrawal, Tolerance, Loss of interest, Loss of control, Role impairment, Concealment, Risky persistence, and Escape coping, respectively. CESD1–CESD3 denote Depressive affect, Positive affect, Somatic symptoms, respectively.


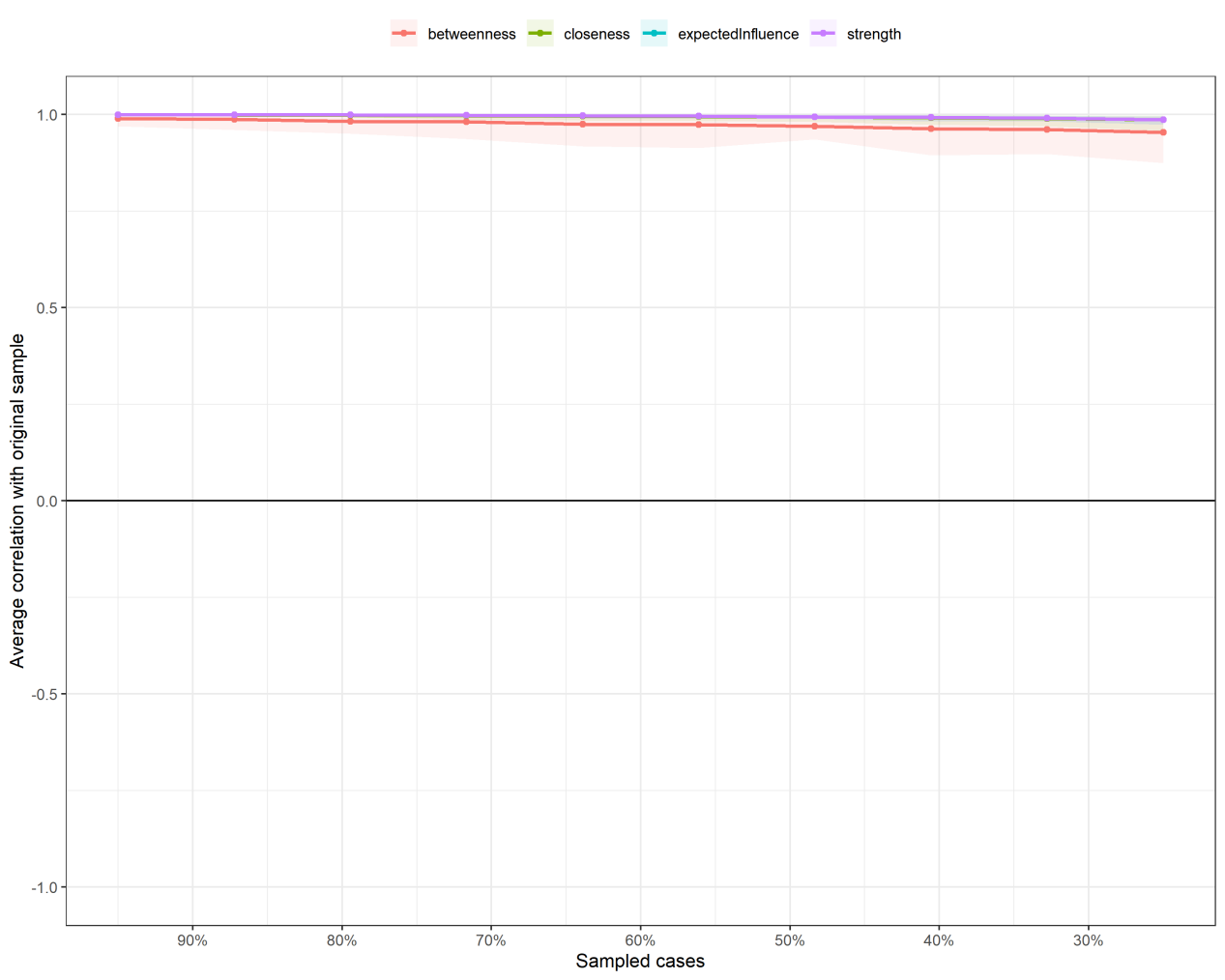


Figure S1.Stability test of network centrality index


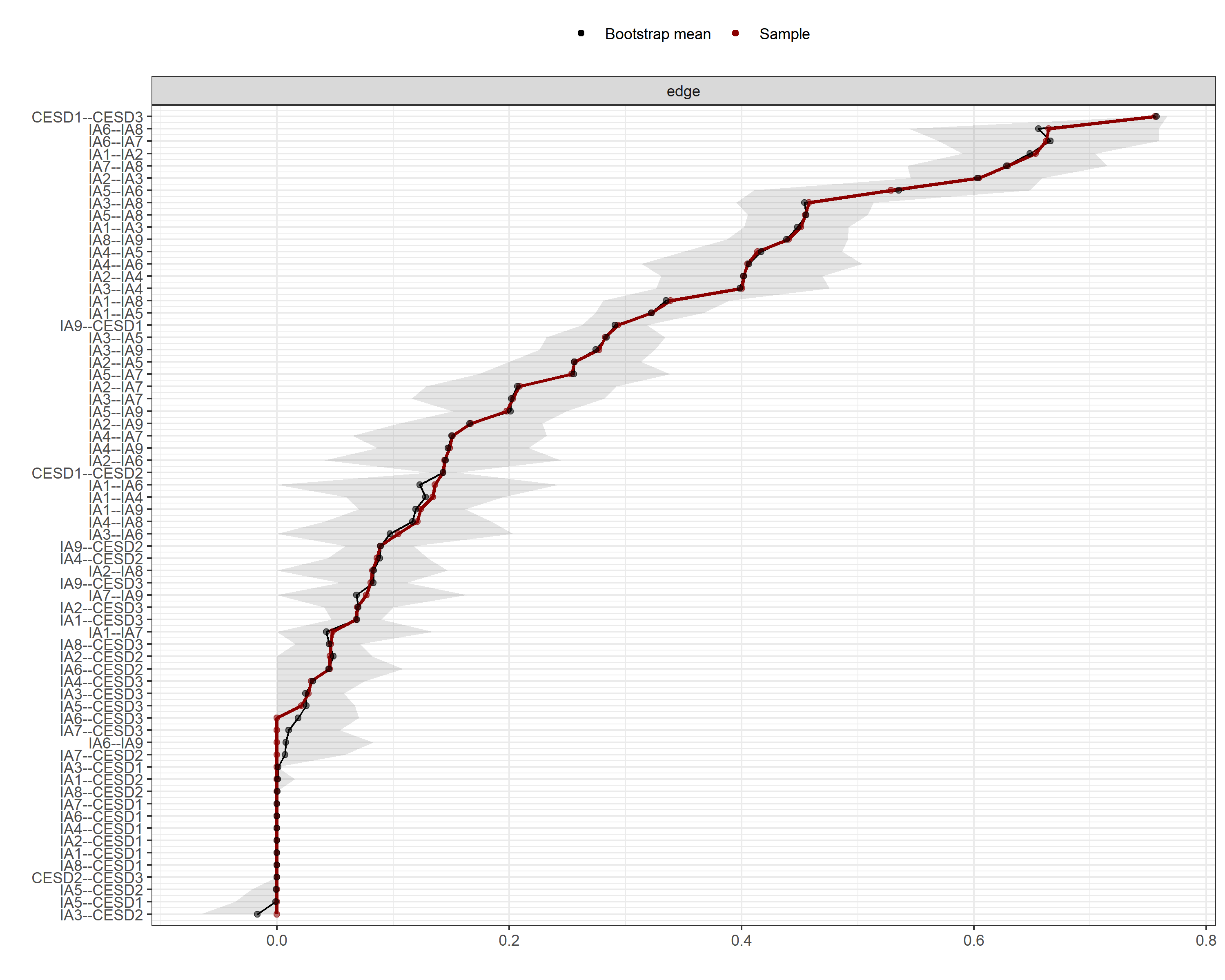


Figure S2.Network edge weight stability test


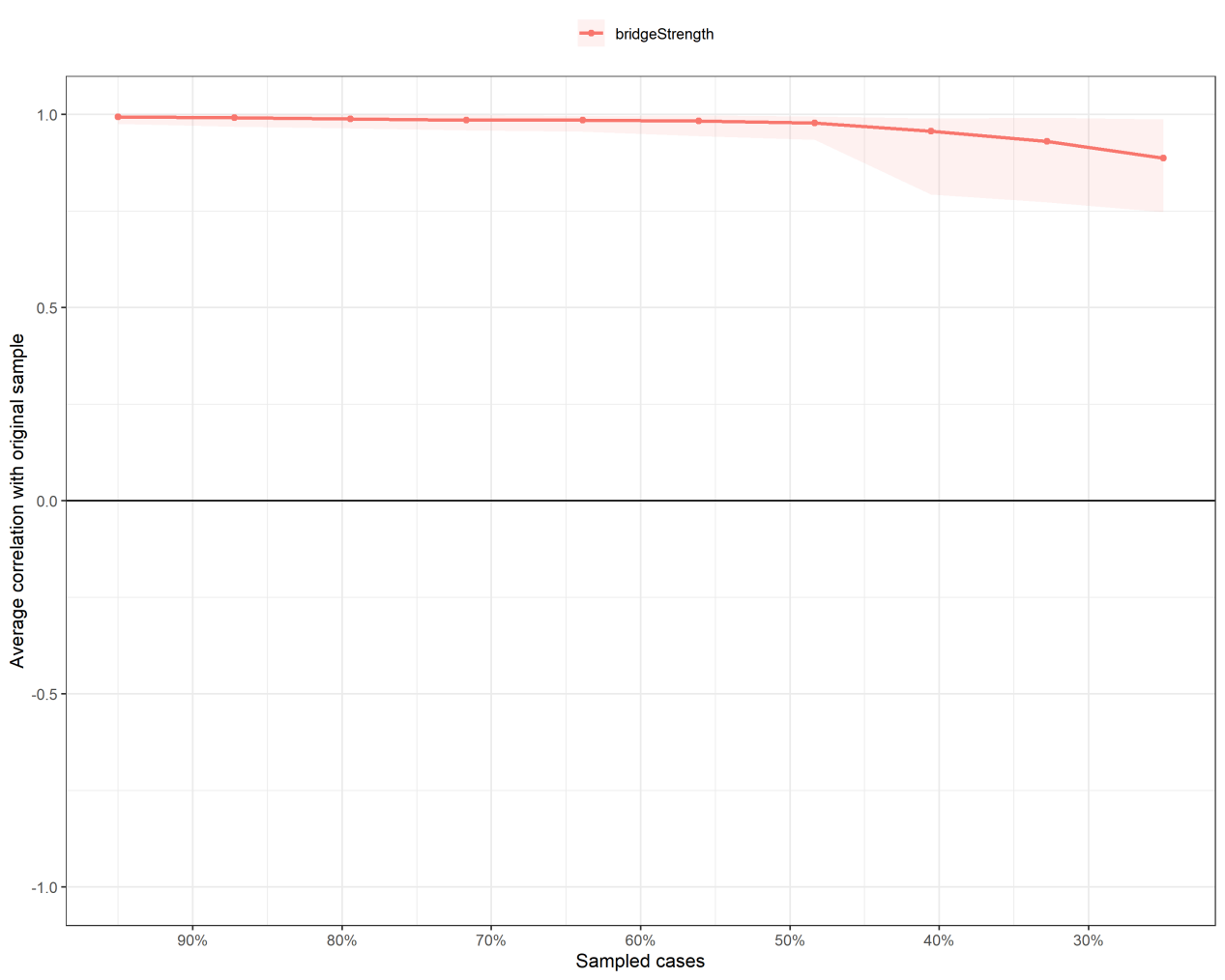


Figure S3.Stability of network bridge strength

Figure S4. Estimated network model for the association between Internet addiction and depression in Left-Behind Children(N=5440) and Non–Left-Behind Children（N=10586）

**A**


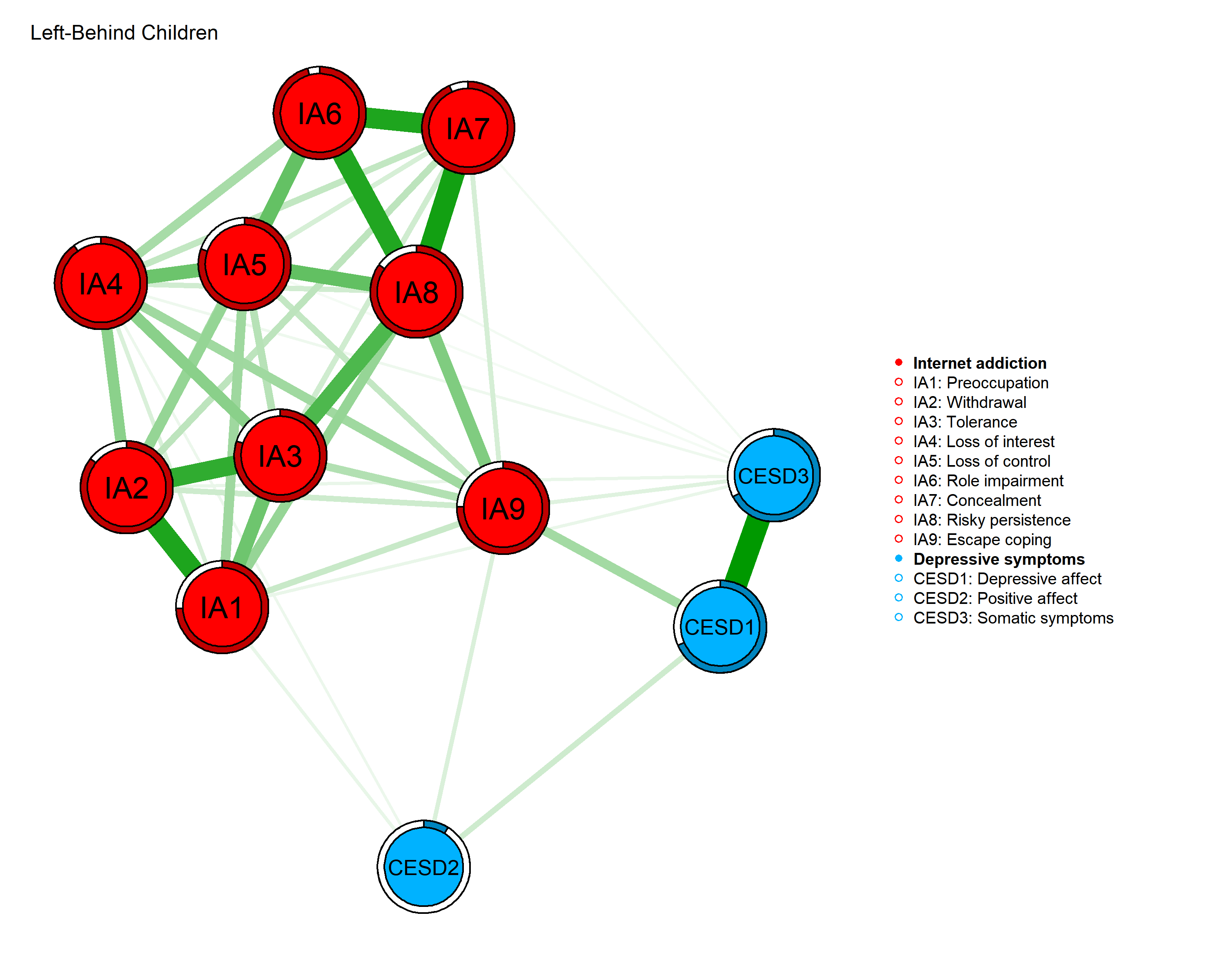


Figure S4.A

**B**


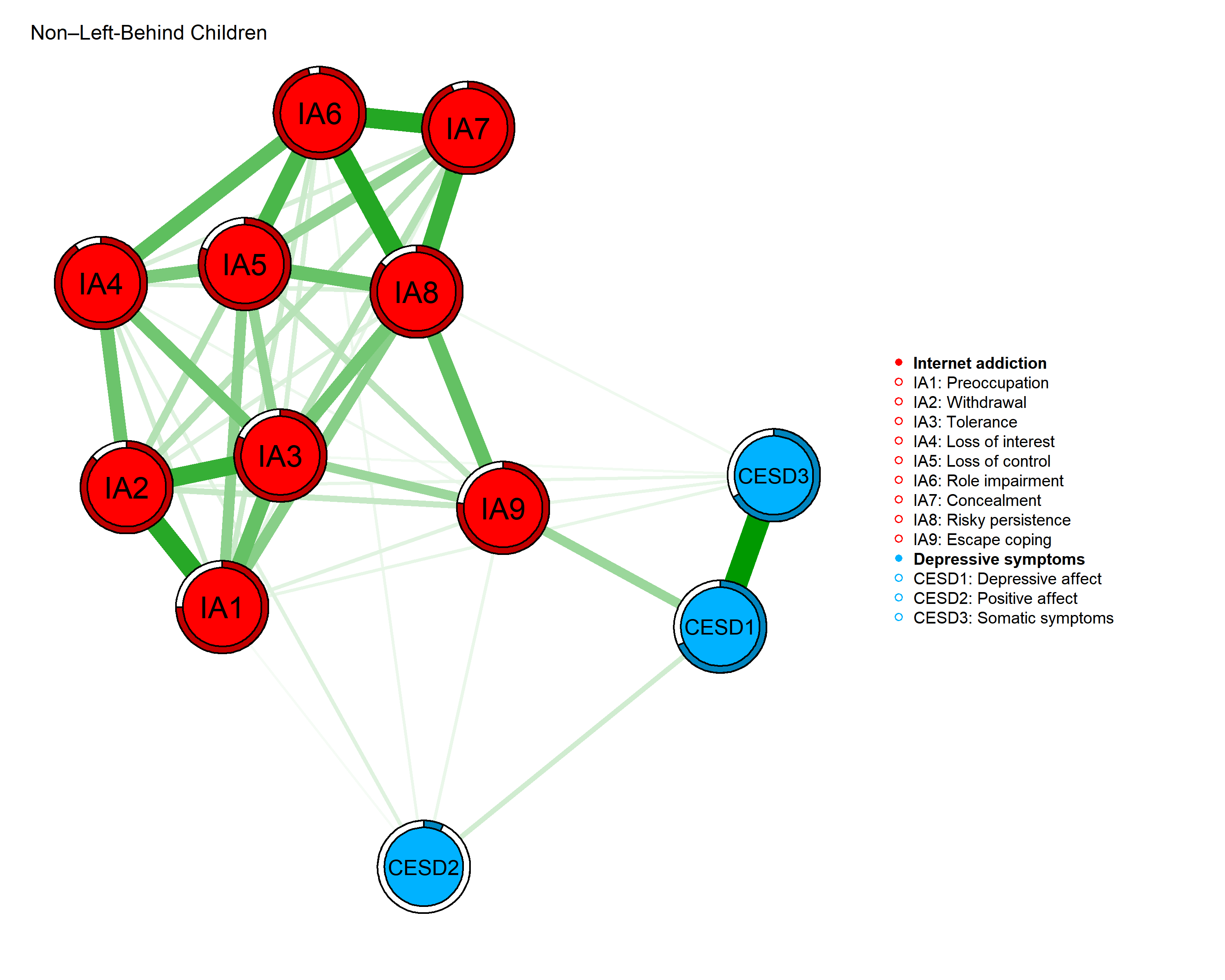


Figure S4.B

**
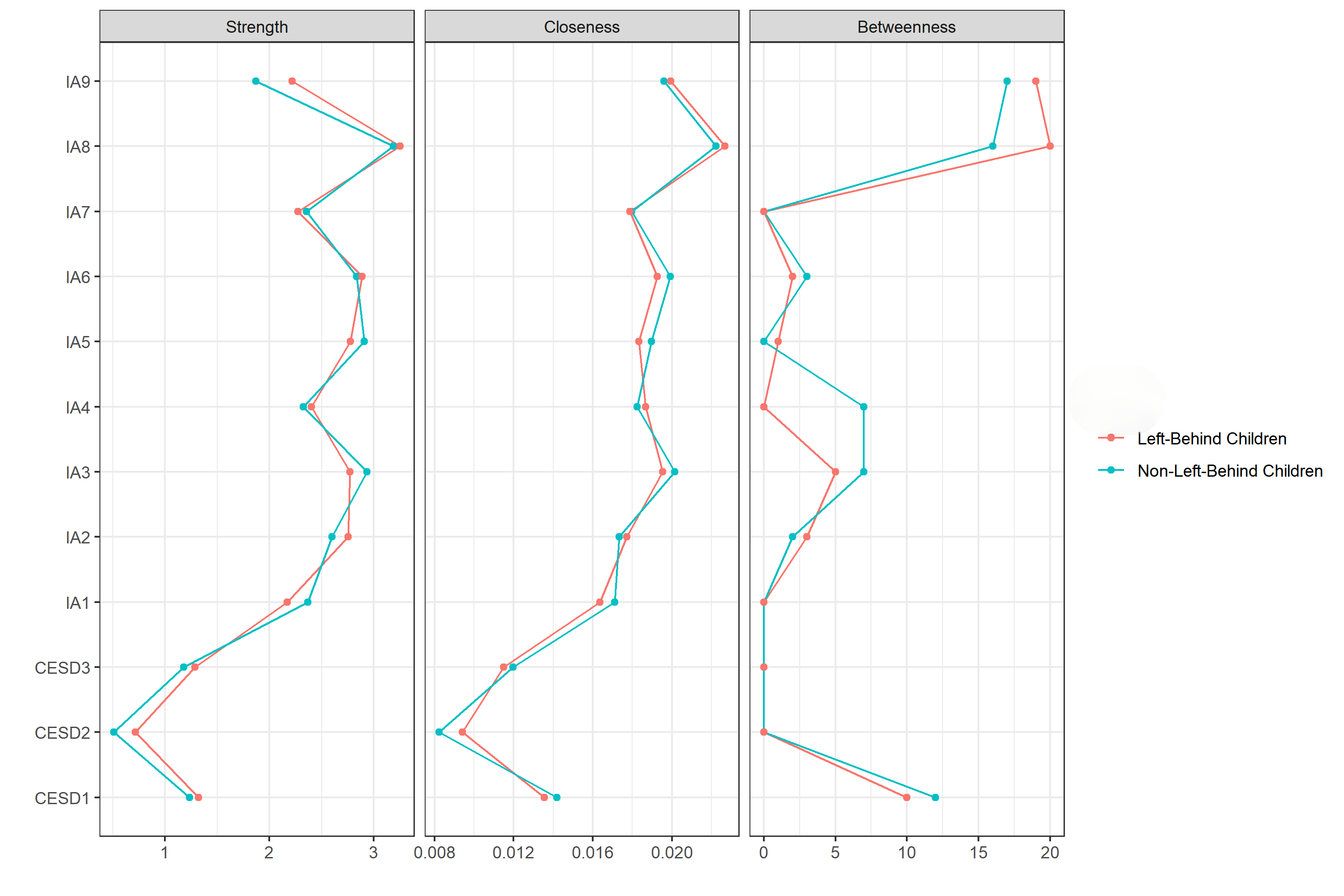
**

**Figure S5. Comparison of network centrality indices between non-left-behind children and left-behind children.**This figure presents the centrality indices of all symptoms in the network (i.e., strength, betweenness, and closeness centrality).
